# Triglyceride-Rich Lipoprotein Modulates Endothelial Vascular Cell Adhesion Molecule (VCAM)-1 Expression via Differential Regulation of Endoplasmic Reticulum Stress

**DOI:** 10.1371/journal.pone.0078322

**Published:** 2013-10-21

**Authors:** Ying I. Wang, Ahmed Bettaieb, Chongxiu Sun, J. Sherrod DeVerse, Christopher E. Radecke, Steven Mathew, Christina M. Edwards, Fawaz G. Haj, Anthony G. Passerini, Scott I. Simon

**Affiliations:** 1 Department of Biomedical Engineering, University of California Davis, Davis, California, United States of America; 2 Department of Nutrition, University of California Davis, Davis, California, United States of America; 3 Department of Internal Medicine, University of California Davis, Davis, California, United States of America; University of Hong Kong, Hong Kong

## Abstract

Circulating triglyceride-rich lipoproteins (TGRL) from hypertriglyceridemic subjects exacerbate endothelial inflammation and promote monocyte infiltration into the arterial wall. We have recently reported that TGRL isolated from human blood after a high-fat meal can elicit a pro- or anti-atherogenic state in human aortic endothelial cells (HAEC), defined as up- or down-regulation of VCAM-1 expression in response to tumor necrosis factor alpha (TNFα) stimulation, respectively. A direct correlation was found between subjects categorized at higher risk for cardiovascular disease based upon serum triglycerides and postprandial production of TGRL particles that increased VCAM-1-dependent monocyte adhesion to inflamed endothelium. To establish how TGRL metabolism is linked to VCAM-1 regulation, we examined endoplasmic reticulum (ER) stress and the unfolded protein response (UPR) pathways. Regardless of its atherogenicity, the rate and extent of TGRL internalization and lipid droplet formation by HAEC were uniform. However, pro-atherogenic TGRL exacerbated ER membrane expansion and stress following TNFα stimulation, whereas anti-atherogenic TGRL ameliorated such effects. Inhibition of ER stress with a chemical chaperone 4-phenylbutyric acid decreased TNFα-induced VCAM-1 expression and abrogated TGRL’s atherogenic effect. Activation of ER stress sensors PKR-like ER-regulated kinase (PERK) and inositol requiring protein 1α (IRE1α), and downstream effectors including eukaryotic initiation factor-2α (eIF2α), spliced X-box-binding protein 1 (sXBP1) and C/EBP homologous protein (CHOP), directly correlated with the atherogenic activity of an individual’s TGRL. Modulation of ER stress sensors also correlated with changes in expression of interferon regulatory factor 1 (IRF-1), a transcription factor of *Vcam-1* responsible for regulation of its expression. Moreover, knockdown studies using siRNA defined a causal relationship between the PERK/eIF2α/CHOP pathway and IRF-1-mediated VCAM-1 expression. We conclude that ER stress and the UPR contribute to the regulation of *Vcam-1* transcription as a function of the atherogenic nature of TGRL.

## Introduction

The composition of circulating triglyceride-rich lipoproteins (TGRL) in blood undergoes dynamic changes with an individual’s diet and lipid metabolism. TGRL consist of proteins and lipids, and their relative amounts and organization determine particle size and density. Particle composition in turn determines its residence time in circulation and the rate of uptake and transport across the endothelium, which directly impact atherogenesis [1]. Hypertriglyceridemia prevails in obese individuals and is directly correlated with the growing epidemic of type 2 diabetes mellitus (T2DM) and increased risk for cardiovascular diseases (CVD) [2]. TGRL from hypertriglyceridemic subjects is thought to exacerbate endothelial inflammation and promote monocyte infiltration into the arterial wall, two early events in the formation of atherosclerotic lesions [3]. Following consumption of a high-fat meal, hypertriglyceridemic subjects produced TGRL particles of larger diameter with increased content of apolipoprotein (Apo)CIII, cholesterol, and triglycerides, and a distinct fatty acid composition [4,5]. Uptake of these TGRL by human aortic endothelial cells (HAEC) amplified upregulation of membrane vascular cell adhesion molecule 1 (VCAM-1), which correlated with an increase in shear resistant monocyte adhesion compared with tumor necrosis factor alpha (TNFα) stimulation alone. Conversely, TGRL with low triglyceride content exerted an athero-protective effect on HAEC, lowering VCAM-1 expression and the extent of monocyte recruitment [4,6,7]. A key question remains as to the origin of this heterogeneity in the inflammatory response to TGRL derived from individuals following a high-fat meal. Specifically, how does uptake and processing of TGRL by the various organelles in endothelial cells (EC) result in differential impact on signaling pathways that converge on a pro- *versus* anti-atherogenic response? 

TGRL and low density remnant particles are promptly taken up by EC via endocytosis through low density lipoprotein (LDL) family receptors, predominantly low density lipoprotein receptor-related protein 1 (LRP-1) and LR11 (LDL receptor relative with 11 binding repeats) [4,8]. Excess fatty acids and sterols released by lipoprotein metabolism are mainly converted to neutral lipids and packaged into lipid droplets (LD) for storage by endoplasmic reticulum (ER). Some metabolic intermediates ultimately enter ER synthesis pathways for phospholipids, a major component of ER and LD membranes [9]. In addition to lipid metabolism, the ER plays a crucial role in protein synthesis [10]. Disruption of ER homeostasis leads to accumulation of unfolded or misfolded proteins in the ER lumen, a condition referred to as ER stress. This condition has emerged as a common signature of metabolic dysregulation as indicated by studies that link obesity to insulin resistance and inflammation in T2DM and CVD [11-13]. A high-fat diet in obese animal models and in obese humans induces marked elevation of ER stress in the liver and adipose tissues, as well as in atherosclerotic lesions [12,14,15]. The mechanism is not entirely known, but is associated with prolonged activation of the ER stress pathway in response to oxidative stress, oxysterols, and elevated levels of intracellular cholesterol and saturated fatty acids in macrophages and endothelial cells [16]. Mitigation of ER stress with the chemical chaperone 4-phenylbutyric acid (4-PBA) has proven effective in alleviating obesity-induced ER stress and insulin resistance in type 2 diabetic mice, and in reducing atherosclerotic lesion progression in high-fat diet fed ApoE^-/-^ mice [17,18]. Still elusive is how the uptake and metabolism of native TGRL exerts a differential inflammatory effect on endothelium and whether ER stress contributes to its atherogenicity. 

Endothelial and other somatic cells have adapted mechanisms to mitigate ER stress and to maintain homeostasis known as the unfolded protein response (UPR), which involves dissociation of binding immunoglobulin protein (BiP) from the three ER transmembrane sensor proteins, PKR-like ER-regulated kinase (PERK), inositol requiring protein 1α (IRE1α), and activating transcription factor 6 (ATF6), and their subsequent activation. These in turn activate downstream effectors, such as spliced X-box-binding protein 1 (sXBP1) and C/EBP homologous protein (CHOP) [19-21]. Apart from being a rescue mechanism under ER stress, some particular UPR pathways play a crucial role in the regulation of adipogenesis and EC inflammation [22,23]. For example, the IRE1α-sXBP1 branch of the UPR mediates interleukin (IL)-8, IL-6, and monocyte chemoattractant protein-1 (MCP-1) expression by HAEC following activation with oxidized LDL [23]. Another subarm of the UPR, the PERK-eukaryotic initiation factor-2α (eIF2α) pathway, is activated in animal models of obesity. Its downstream target CHOP is highly expressed in atherosclerotic plaques and reported to mediate ER stress-induced upregulation of inflammation and adhesion related proteins in macrophages and smooth muscle cells, in addition to its traditional pro-apoptotic role [24,25]. Thus, a constellation of evidence points to UPR and ER stress being directly associated with the inflammatory axis of atherogenesis. Yet it is unknown whether and to what extent they are engaged in the modulation of endothelial inflammation as a function of an individual’s TGRL.

We hypothesized that the relative capacity of TGRL to influence transcription and expression of VCAM-1 is a function of particle metabolism, in particular its influence on ER stress. Our objective was to investigate a role for ER stress in TGRL-modulated endothelial dysfunction using a model for hypertriglyceridemia. To that end, we assessed TGRL harvested from low- and high-risk subjects (e.g. based upon serum triglyceride level) fed a high-fat meal and examined ER stress and UPR signaling pathways in the transcriptional regulation of VCAM-1 expression following EC inflammation stimulated by TNFα. We demonstrate that native TGRL edits inflammatory VCAM-1 expression via UPR pathways.

## Materials and Methods

### Human subjects and study meal

Volunteers were recruited under informed written consent according to a protocol approved by the Institutional Review Board at the University of California, Davis. The study included both normal subjects and individuals with hypertriglyceridemia (fasting serum triglycerides > 150 mg/dL), and excluded those on lipid-lowering or anti-inflammatory medications or with a fasting blood glucose > 110 mg/dL. Venous blood was collected after a 12-hour overnight fast and again 3.5 hours after a standardized moderately high-fat meal (1230 calories, 47% from fat; 32% of total fat is saturated fat) [4]. Fasting and postprandial serum lipid and glucose levels were determined by the UC Davis Medical Center Clinical Laboratory using the Beckman Coulter UniCel DxC 800 Synchron Clinical System. LDL cholesterol was calculated by the Friedewald equation [26].

### TGRL isolation and characterization

TGRL (ρ < 1.0063 g/ml) were isolated from postprandial blood samples by ultracentrifugation, and normalized for ApoB content quantified by ELISA (Alerchek), as previously described [8]. The isolates tested negative for endotoxin by chromogenic test kit (Associates of Cape Cod). They were aliquoted under nitrogen at 4°C for immediate use. Particle size distribution was measured using a Nanotrac Particle Size Analyzer equipped with FLEX software (Microtrac Inc.) as described previously [4]. More than 95% of the isolated TGRL particles were categorized as very-low-density lipoproteins (VLDL) based on particle diameter. For each subject’s sample, lipid and apolipoprotein concentrations (total cholesterol, triglycerides, ApoB, ApoCIII and ApoE) were measured by spectrophotometric assay using a clinical chemistry analyzer (Polymedco Inc.). 

### HAEC culture and TGRL treatment protocol

Low passage HAEC (passage 4-6) (Genlantis) were cultured on collagen-coated (50µg/ml, Clontech) culture dishes or gelatin-coated (1%) coverslips and maintained in Endothelial Cell Growth Medium-2 (EGM^®^-2, Lonza) supplemented with 1x antibiotic-antimycotic solution (Invitrogen). Unless otherwise indicated, HAEC were treated with TNFα (0.3 ng/ml, R&D system) alone or simultaneously with individual freshly isolated TGRL (10 mg ApoB/dL) for 4 hours. To quantify inflammatory activation relative to TNFα alone, HAEC were detached using enzyme-free cell dissociation buffer (Gibco), labeled with fluorescein-conjugated antibodies to human intercellular adhesion molecule 1 (ICAM-1) or VCAM-1, and analyzed by a FACScan flow cytometer (Becton Dickinson) [4]. Median fluorescence intensity (MFI) was quantified using FlowJo software (TreeStar, Ashland, OR) and used for further analysis.

### Antibodies

For flow cytometry, the following antibodies from R&D systems were used: hVCAM-1 FITC mAb (BBA22), hICAM-1 FITC mAb (BBA20), and IgG fluorescein isotype controls (IC002F and IC003F). For Western blotting, the following antibodies were used: BiP (ab21685), phospho-IRE1α (ab48187), and IRF-1 (ab55330) from Abcam; total IRE1α (3294), total-eIF2α (2103), phospho-eIF2α (3597), CHOP (5554) from Cell Signaling Technology; sXBP1 (sc-32138) and Tubulin (sc-53646) from Santa Cruz Biotechnology; and GAPDH (NB-300-328) from Novus Biologicals. For immunofluorescence staining of ER structure, goat anti-human calreticulin antibodies (SC-6468) from Santa Cruz Biotechnology and Alexa Fluor^®^ 546 rabbit anti-goat IgG (A-21085) from Life Technologies were used.

### TGRL labeling and uptake assay

TGRL were conjugated to Alexa-Fluor 488 (AF488) using a protein labeling kit (Invitrogen) as described previously [4], and stored at 4°C, protected from light. For the uptake assay, HAEC were incubated with AF488-TGRL (10 mg ApoB/dL in phenol red-free culture media) at 37°C for the designated period, washed twice with Hank's Balanced Salt Solution (HBSS, w/Ca^2+^, Life Technologies), detached using TrypLE Express (Life Technologies), pelleted at 1,000 g at 4°C, and resuspended in HBSS (w/Ca^2+^ + 0.2% human serum albumin) on ice. The samples were immediately analyzed by a FACScan flow cytometer (Becton Dickinson) with CellQuest software. Data represent MFI from a single Gaussian population of 10,000 cells for each sample.

### Lipid droplet staining and imaging

HAEC were incubated with TGRL (10 mg ApoB/dL) for the indicated period, washed with 37°C HBSS w/ Ca^2+^, and incubated with BODIPY 493/503 (Life Technologies, 2 µM in HBSS) at 37°C for 1 min. HAEC were washed with ice cold HBSS, collected using TrypLE Express, and analyzed by a FACScan flow cytometer. Samples were protected from light throughout these procedures. Data represent MFI from a single Gaussian population of 10,000 cells for each sample. For visualization of LD by confocal microscopy, HAEC were cultured on 1% gelatin-coated coverslips, incubated with TGRL for 4 hours, and stained with BODIPY as described above. Cells were maintained in phenol red-free Leibovitz-15 medium (Life Technologies) at 37°C, and imaged under 488nm laser excitation by a Zeiss LSM 5 Pascal confocal microscope.

### Immunofluorescence

HAEC were grown on 1% gelatin-coated coverslips to 80% confluence. After treatment, cells were rinsed with phosphate-buffered saline (PBS), fixed and permeabilized with 100% methanol at -20°C for 6 min. The coverslips were then rinsed with PBS and blocked in 5% bovine serum albumin (BSA) + 0.2% Triton X-100 in PBS. HAEC were then incubated with goat anti-human calreticulin antibody (1 µg/ml) in PBS w/ 1% BSA for 1 hour at room temperature, washed with PBS, and incubated with Alexa Fluor 546 rabbit anti-goat IgG (2 µg/ml) in PBS at room temperature for 1 hour. Nuclei were counterstained with Hoechst (Sigma). The samples were imaged using a Zeiss LSM 5 Pascal confocal microscope. Images were analyzed using ImageJ. The coefficient of variation (CV) in the pixel fluorescence intensity of labeled calreticulin in the cytoplasm of each cell, a global index of spatial heterogeneity, was applied to assess ER morphological changes. Cells with expanded ER yielded higher CV values as calreticulin became less evenly distributed [27]. 30-40 cells were analyzed for each condition.

### RNA isolation and quantitative RT-PCR

Total RNA was isolated using the High Pure Total RNA isolation kit (Roche), which includes a DNase digestion step to remove contaminating genomic DNA. RNA (~500 ng) was converted to 1st strand cDNA using the Transcriptor First Strand cDNA Synthesis kit (Roche). Quantitative PCR was performed using TaqMan Gene Expression Assays (Applied Biosystems, VCAM-1: Hs00365485_m1; ICAM-1: Hs00164932_m1; RPLP0: Hs99999902_m1; IRF-1: Hs00971960_m1) and TaqMan Gene Expression Master Mix (Applied Biosystems) with a RealPlex Mastercycler (Eppendorf). Quantification relative to the housekeeping gene acidic ribosomal protein P0 (RPLP0) was determined by the ΔΔC_t_ method, where C_t_ is threshold cycle number. 

### Protein extraction and Western immunoblot analysis

Cells were lysed in radio immunoprecipitation assay (RIPA) buffer containing 1 mM NaF, 1 mM sodium orthovanadate and protease inhibitor cocktail (Sigma) with freshly added 0.1 mM phenylmethanesulfonylfluoride (PMSF). The lysate was sonicated and protein concentration determined by the bicinchoninic acid (BCA) assay. Samples were normalized to 1 mg/ml of protein content, and resolved by SDS-PAGE, transferred to nitrocellulose, and blocked. The blots were incubated overnight at 4°C with primary antibody, then at room temperature for 1.5 hours with horseradish peroxidase-conjugated secondary antibody. They were developed with Chemiluminescent Substrate. Signals were captured by ChemiDoc MP gel imaging system (Bio-Rad). Tubulin, GAPDH or the total protein forms of phosphorylated proteins of interest served as the loading control for quantification using ImageJ [5].

### Cell Transfection

To deplete CHOP or sXBP1 expression, cells (5×10^5^) were transfected with 150 pmol siRNA to CHOP or XBP1 (Santa Cruz Biotech), using a 4D-Nucleofector system (Lonza). After 48-96 hr, cells were treated as described followed by Western blot or flow cytometry analysis.

### NF-κB and activator protein-1 (AP-1) activation

NF-κB and AP-1 activities were measured by ELISA targeting active phospho-p65 and phospho-c-Jun in the nuclear extract as previously described [5].

### Statistical analysis

Data were analyzed using GraphPad Prism v5.0 software. All data are presented as mean ± standard error (SE) unless otherwise indicated. Differences among multiple groups were assessed by one-way ANOVA with a post hoc Newman-Keuls test. Two experimental groups were compared using Student’s *t* test, with pairing where appropriate. Comparisons were considered significant for 2-tailed p values < 0.05. Correlations between groups were assessed using Pearson's correlation coefficient (r).

## Results

### TGRL uptake induces extensive lipid droplet formation independent of its atherogenicity

To investigate the origin of the heterogeneity in the atherogenicity of TGRL isolated from blood collected following consumption of a high-fat meal, we recruited thirty-four subjects with normal fasting glucose, but exhibiting a broad range in anthropometric characteristics and fasting serum triglycerides from normal to hypertriglyceridemic (Tables S1 and S2). We categorized each subject’s postprandial TGRL based on its capacity to alter the inflammatory response of HAEC stimulated with TNFα, a pivotal inflammatory cytokine expressed locally at sites of atherosclerotic plaques [28] and a biomarker for CVD that is found at elevated levels in the serum of individuals at increased risk [29]. The dose of TNFα in use (0.3 ng/ml) was previously reported to correspond to the EC_50_ for upregulation of membrane adhesion receptors on HAEC (VCAM-1, ICAM-1, or E-selectin) [8]. TGRL that enhanced VCAM-1 expression relative to TNFα stimulation alone was designated as pro-atherogenic and that which suppressed VCAM-1 expression as anti-atherogenic [5,16]. Pro-atherogenic TGRL were enriched in triglyceride, cholesterol, ApoE, and ApoCIII content per particle as compared to anti-atherogenic that uniformly expressed lower levels (Figure S1).

**Figure 1:**
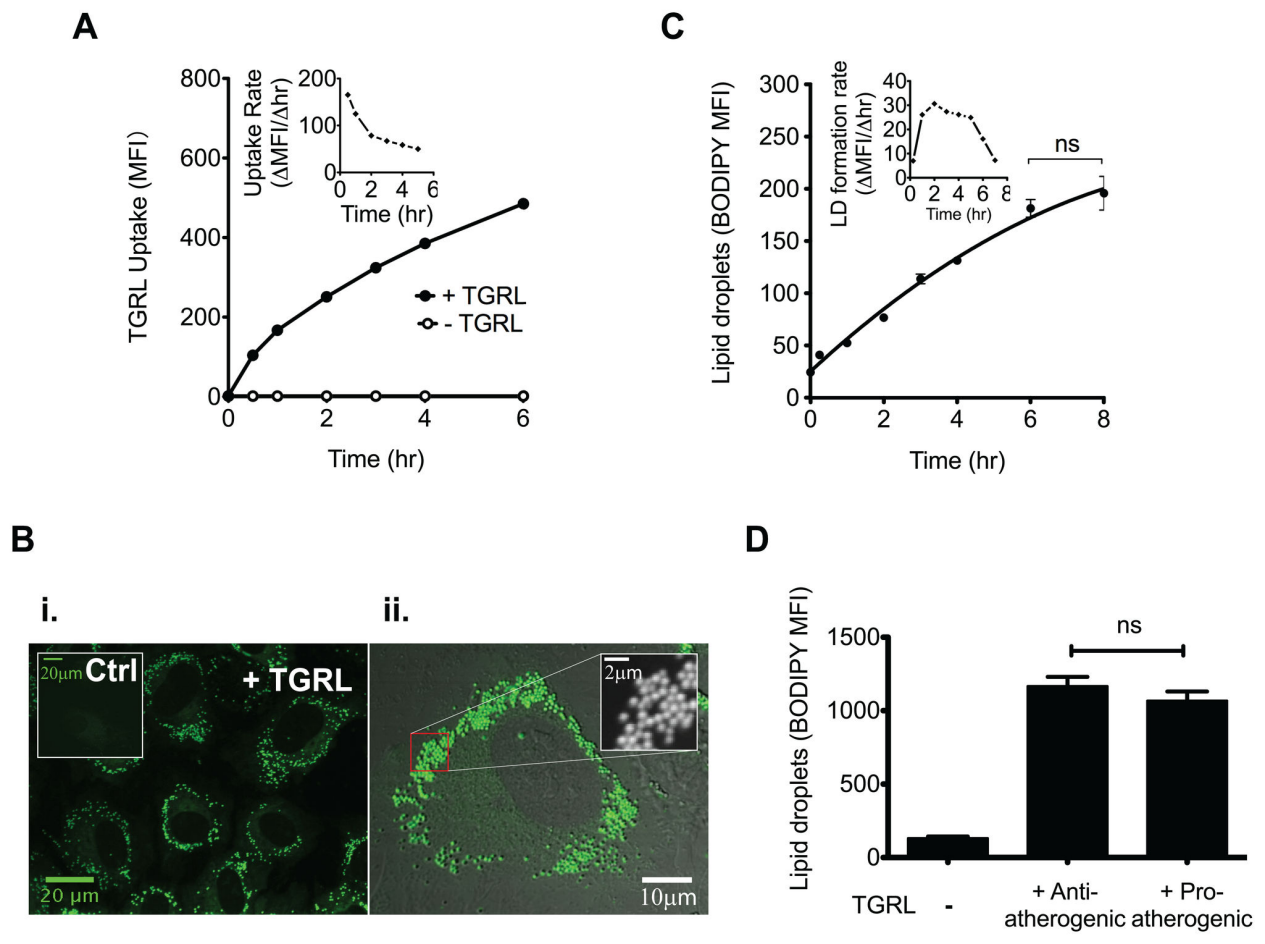
TGRL induces extensive lipid uptake and lipid droplet formation. (A) The kinetics of TGRL uptake. HAEC were incubated in the presence (“●”) or absence (“○”) of Alexa fluor488-labeled TGRL (10 mg ApoB/dL) for 0-6 hr, and examined for internalized TGRL by flow cytometry. Data presented as median fluorescence intensity (MFI). Inset: uptake rate defined as ∆MFI/∆hr. (B) Representative confocal images (i. and zoomed-in version ii.) of lipid droplets formed in HAEC. Cells were treated for 4 hr with native TGRL (10 mg ApoB/dL) or culture media (“Ctrl”), and stained for lipid droplets with BODIPY 493/503. (C) The kinetics of lipid droplet formation. HAEC were incubated with native TGRL for designated periods, stained with BODIPY and analyzed by flow cytometry. n=3. Inset: the rate of lipid droplet formation = ∆MFI of BODIPY/∆hr. (D) Quantification of lipid droplets formed in HAEC after 4 hr incubation with anti- or pro-atherogenic TGRL. n=3-6. ns, not significant.

We next investigated whether the differential atherogenic response of HAEC to inflammatory stimulation was a function of the extent of TGRL uptake and subsequent lipid droplet (LD) formation. The internalization of fluorophore-conjugated TGRL particles increased rapidly upon addition to the EC monolayer and rose steadily up to 6 hr (Figure 1A), while the uptake rate reached a steady state after 2 hr (Figure 1A, inset). Intracellular LD formation was visualized by confocal fluorescence microscopy of BODIPY stained EC (Figure 1B). Discrete punctate BODIPY-positive droplets measuring ~0.1-0.5 micrometer in diameter were resolved in HAEC cytoplasm. To determine whether the rate and extent of LD formation correlated with TGRL atherogenicity, we quantified their cytoplasmic abundance using flow cytometry. LD formation increased with a time course similar to that of TGRL uptake at a rate that plateaued at 2 hr and maintained a steady state up to 5 hr (Figure 1C, inset). LD abundance in TGRL treated HAEC was increased by ~6-fold at 4 hr with no significant difference observed in the extent of LD formation between anti- and pro-atherogenic TGRL (Figure 1D), which decreased VCAM-1 expression by 10 ± 2 %, and increased it by 29 ± 7 %, respectively, compared to TNFα alone. Noteworthy was the observation that the extent of LD formation as measured by BODIPY fluorescence correlated closely with the side scatter (SSC) signal obtained by flow cytometry (r = 0.94, P < 0.0001, Figure S2), suggesting that SSC provides a simple label-free approach to assess LD abundance in EC. Together, these data demonstrate a rapid and sustained TGRL uptake that results in the extensive formation of LD in the cytoplasm of HAEC. Heterogeneity of particle size and composition that discriminated anti- from pro-atherogenic TGRL did not result in a discernible difference in the extent of LD formation. We conclude that the relative atherogenicity of TGRL on HAEC is not simply a function of its relative abundance or extent of cytoplasmic internalization.

### TGRL atherogenicity is reflected in ER morphological changes associated with ER stress

**Figure 2:**
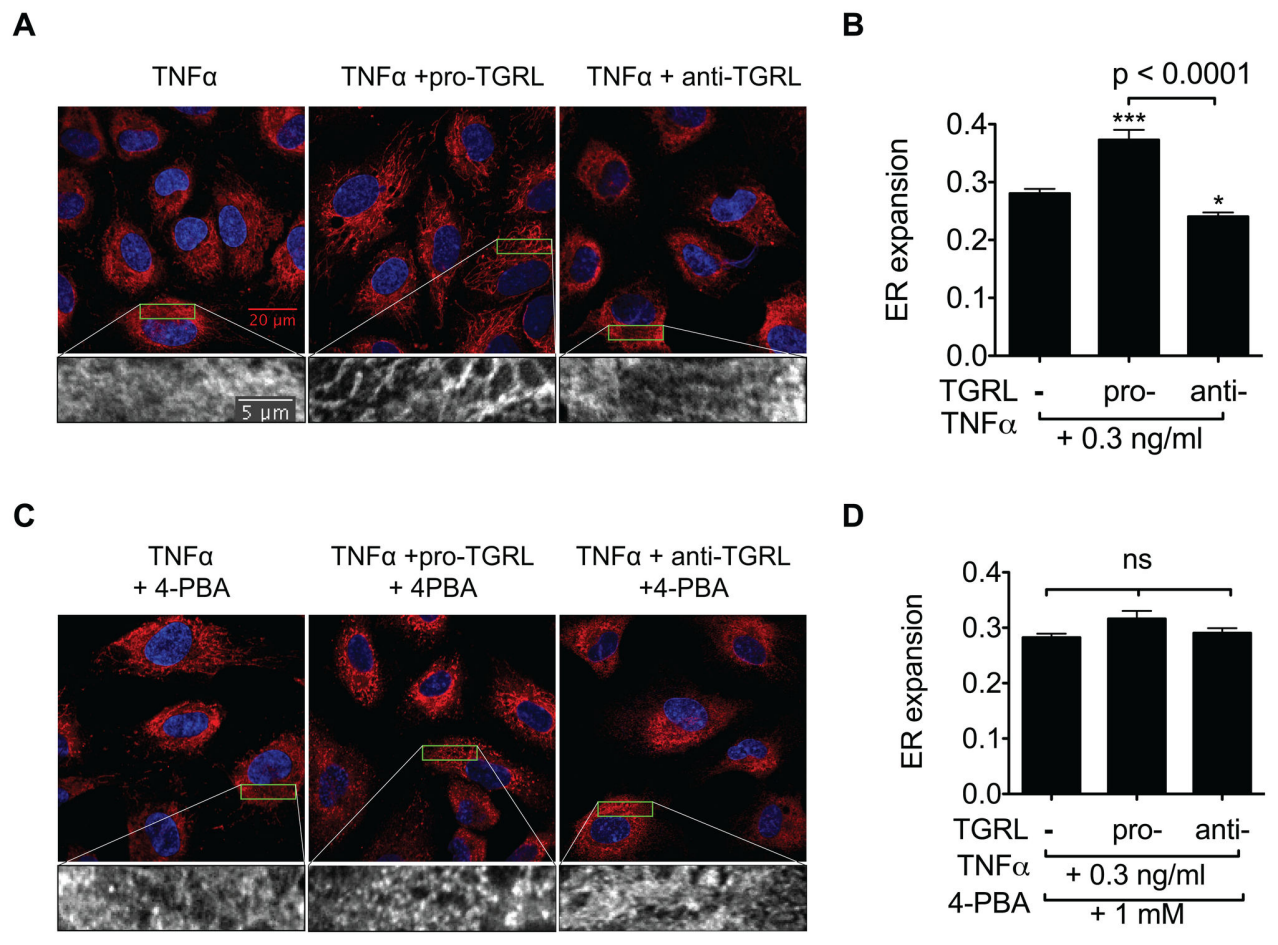
TGRL-induced ER morphological changes are alleviated by an ER stress inhibitor. A-B: Pro-atherogenic TGRL induces ER expansion, defined as increased spatial heterogeneity (Standard Deviation/Mean of pixel fluorescence intensity within cell cytoplasm), while anti-atherogenic TGRL suppresses it. HAEC grown on coverslips were treated for 4 hr with TNFα alone or simultaneously with pro- or anti-atherogenic TGRL (10 mg ApoB/dL). Cells were then visualized by indirect immunofluorescence and confocal microscopy. Representative confocal images shown in (A) red, calreticulin; blue, Hoechst nuclear stain. Bottom panels show zoomed images of calreticulin signal from the boxed 20µm × 5µm region. Statistical analysis of ER expansion shown in (B). 30-40 cells were analyzed for each condition. ***P<0.001, *P<0.05 from TNFα-stimulated. C-D: ER stress inhibition normalizes TGRL’s effect on ER morphology. 4-phenyl butyric acid (4-PBA) was added to the above treatments with additional 1 hr pretreatment. Confocal images shown in (C) and statistical analysis in (D). 30-40 cells were analyzed for each condition. ns, not significant.

Excess fatty acids and sterols released from lipoproteins are mainly converted to neutral lipids and stored in LD by a series of ER-resident enzymes [9]. We next examined whether the atherogenic phenotype induced by uptake and metabolism of individual TGRL was reflected in alteration of ER [9]. One readout of ER dysfunction is distortion in the composition and structure of its membrane, as previously observed in smooth muscle cells [30] and livers of obese mice fed diets high in fat [31]. To investigate if the metabolic challenge induced by TGRL uptake and subsequent LD formation effectively disrupts ER function, we examined ER morphology after 4 hr incubation with pro- or anti-atherogenic TGRL or control media in the presence of TNFα stimulation. HAEC ER morphology was imaged by confocal microscopy and immunofluorescence staining of calreticulin, an ER luminal resident protein [32]. Image analysis revealed a significant extent of ER membrane expansion in HAEC treated with pro-atherogenic TGRL (Figure 2A), similar to that previously reported following skeletal muscle uptake of saturated fatty acid [30]. ER membrane expansion was characterized by an increase in tubular and web structures with a significant 33% increase in the spatial heterogeneity of labeled calreticulin in HAEC treated with pro-atherogenic TGRL and stimulated with TNFα (Figure 2B). In contrast, anti-atherogenic TGRL suppressed ER expansion by 14%. To test whether ER stress was associated with the differential effects of pro- *versus* anti-atherogenic TGRL, we examined ER membrane alteration in the presence of the ER stress inhibitor, 4-phenyl butyric acid (4-PBA). Treatment with 4-PBA abrogated the effect of pro-atherogenic TGRL on ER expansion (Figure 2C-D). Further, we confirmed that TNFα itself did not cause obvious morphological changes compared to control cells; however, treatment of HAEC with pro-atherogenic TGRL alone induced a significant increase in ER expansion that was augmented upon TNFα stimulation (Figure S3). These data demonstrate that ER membrane morphology provides a reliable readout of the relative effects of TGRL and suggest that ER stress may directly influence downstream inflammatory responses of HAEC. 

### ER stress plays a key role in TNFα and TGRL modulation of VCAM-1 expression

**Figure 3:**
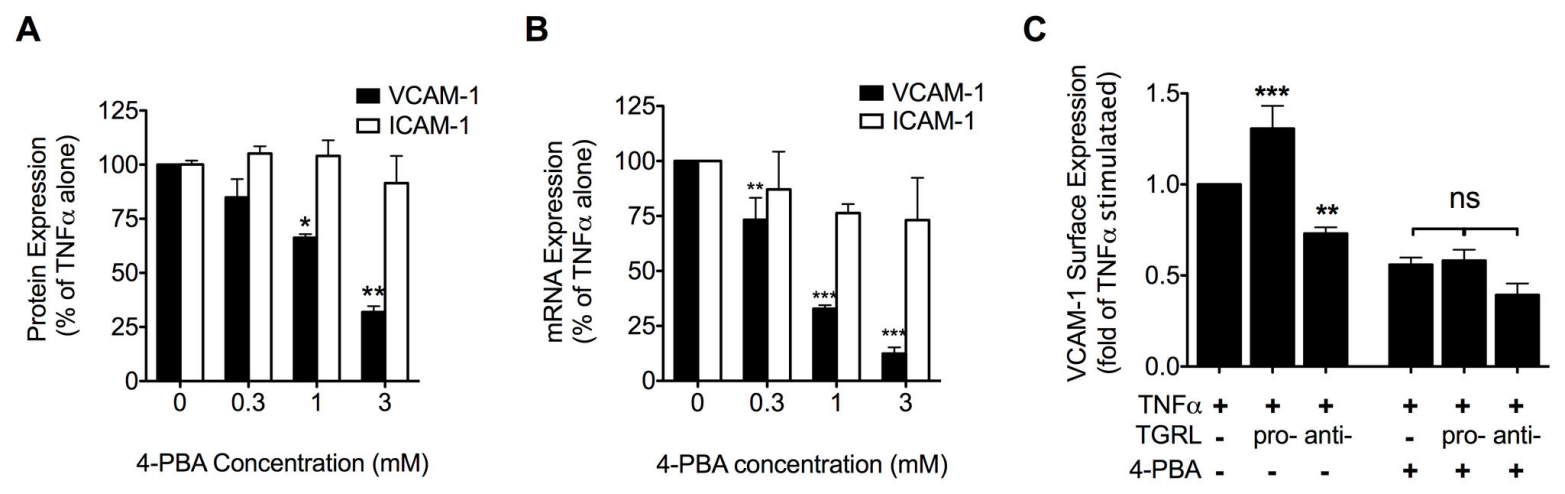
ER stress plays a key role in TGRL modulation of TNFα-stimulated VCAM-1 expression. A-B: ER stress inhibition dose-dependently reduced TNFα-induced VCAM-1 expression but did not affect ICAM-1 expression. HAEC were conditioned for 4 hr (A) or 2 hr (B) with TNFα (0.3 ng/ml) and 4-phenyl butyric acid (4-PBA) at different doses (0, 0.3, 1, or 3 mM, with 1 hr pretreatment). VCAM-1 and ICAM-1 protein or mRNA expression analyzed by flow cytometry (A) or quantitative RT-PCR (B). n=3-5. *P<0.05, **P<0.01 from TNFα stimulated alone. (C) ER stress inhibition abolished TGRL modulation of TNFα-stimulated VCAM-1 expression. HAEC were treated for 4 hr with TNFα (0.3 ng/ml) alone or simultaneously with pro- or anti-atherogenic TGRL (10 mg ApoB/dL) in the presence (1 mM, 1 hr pretreatment and 4 hr co-treatment) or absence of 4-PBA. n=4-7. ***P<0.001, **P<0.01 from TNFα-stimulated alone. ns, not significant.

The distinct alterations of ER morphology by pro- and anti-atherogenic TGRL led us to further investigate whether inhibition of ER stress with 4-PBA would alter TNFα-stimulated upregulation of adhesion receptor expression. Treatment of HAEC with 4-PBA significantly inhibited VCAM-1 upregulation in a dose-dependent manner, yielding an IC_50_ of 2mM and ~70% inhibition at 3mM 4-PBA following 4 hrs of stimulation (Figure 3A and Figure S4 A-B). In contrast, 4-PBA did not affect ICAM-1 upregulation within this dose range (Figure 3A and Figure S4 C-D). Similar results were observed for regulation of mRNA production at the 2 hr time point (Figure 3B). This suggested that ER stress was modulating a VCAM-1-specific regulatory pathway. To determine whether inhibition of ER stress also modulated the atherogenic effect of TGRL on VCAM-1 expression, HAEC were treated with pro- or anti-atherogenic TGRL and stimulated with TNFα in the presence or absence of 4-PBA. Suppression of ER stress by 4-PBA abrogated the pro-atherogenic effect of TGRL in regulating TNFα-stimulated VCAM-1 expression (Figure 3C). These results clearly implicate ER stress in TNFα and TGRL-modulated adhesion molecule upregulation.

### TGRL modulates VCAM-1 expression through activation of ER stress

**Figure 4:**
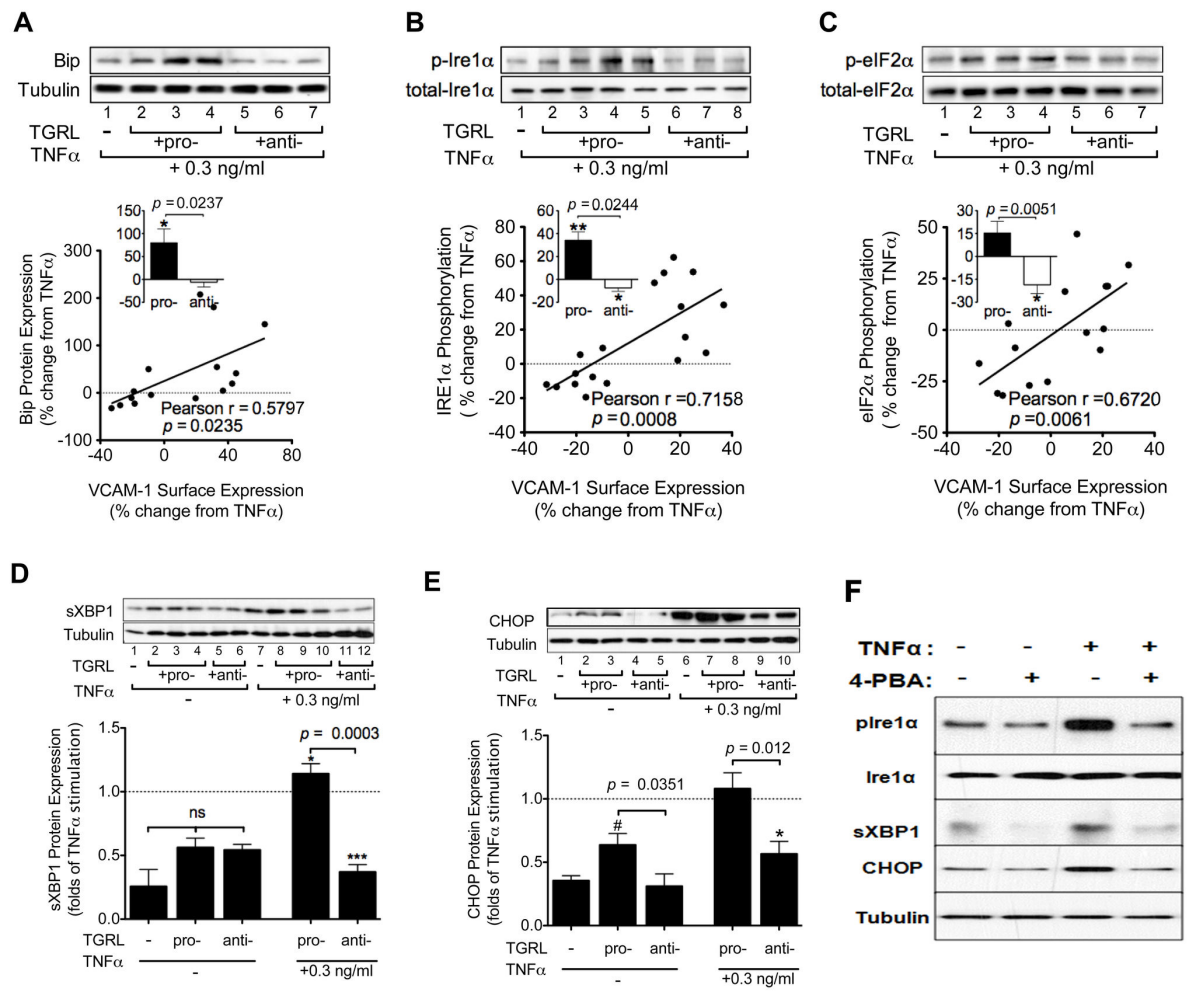
TGRL modulates ER stress markers as a function of its atherogenicity. HAEC were treated for 4 hr with TNFα alone or simultaneously with TGRL (10 mg ApoB/dL). Protein extracts from whole cell lysates were analyzed by Western immunoblot. Representative blots are shown. A-C: Protein expression level or phosphorylation ratio presented as % change from TNFα-stimulated alone. Pearson correlations between TGRL modulation of TNFα-induced VCAM-1 expression and (A) BiP expression, (B) IRE1α or (C) eIF2α phosphorylation. n = 15-18. Insets: the same data binned into two categories based on TGRL’s atherogenicity. *P<0.05, **P<0.01 from TNFα. D-E: Pro-atherogenic TGRL enhanced (D) sXBP1 expression, while anti-atherogenic TGRL suppressed (D) sXBP1 and (E) CHOP expression. ns, not significant. *P<0.05, ***P<0.001 from TNFα-stimulated. #P<0.05 from non-stimulated control. n=3-9. (F) HAEC were treated for 4 hr with TNFα (0.3 ng/ml) with or without pretreatment with 1 mM 4-PBA, followed by Western blot analysis, representative of 3 separate experiments.

We next investigated the ER stress signaling pathways that may contribute to the atherogenic effect of TGRL specifically on VCAM-1 expression. We first measured changes in BiP protein expression and the phosphorylation of IRE1α (Ser^724^) and eIF2α (Ser^51^), adjusted for their relative levels of protein expression. Western immunoblotting revealed that TNFα at 0.3 ng/ml elicited 80% upregulation of BiP expression and 50% of IRE1α phosphorylation over baseline, which equals 42% and 58% of the elevation by TNFα at 10 ng/ml (Figure S5). Pro-atherogenic TGRL on average upregulated BiP expression by ~80% and increased the phosphorylation of both IRE1α (35%) and eIF2α (15%) from the modest effect of TNFα alone, whereas anti-atherogenic TGRL had comparatively less of an effect on BiP and IRE1α (Figure 4A-C). Further, a significant direct correlation was observed in activity of all three ER stress markers *versus* regulation of VCAM-1 expression elicited by treatment with each subject’s TGRL relative to TNFα alone. To better understand the effect of TGRL on downstream mediators of ER stress we assessed activation of sXBP1 and CHOP. Baseline expression of sXBP1 protein was detected at low levels and not significantly elevated by treatment with TGRL alone. TNFα stimulation significantly increased the level of sXBP1, which was further elevated by pro-atherogenic TGRL (15%) and decreased by anti-atherogenic TGRL (63%) relative to TNFα stimulation alone (Figure 4D). In contrast, baseline expression of CHOP was increased by 80% following treatment with pro-atherogenic TGRL, but remained unchanged in response to anti-atherogenic TGRL. TNFα stimulation alone increased CHOP expression by 3-fold and this rise was suppressed by anti-atherogenic TGRL (Figure 4E). Both sXBP1 and CHOP levels significantly correlated with regulation of VCAM-1 expression elicited by treatment with each subject’s TGRL compared to TNFα alone (Figure S6). As expected, inhibition with 4-PBA down regulated cytokine activation of ER stress as indicated by an attenuated phosphorylation of IRE1α and reduced expression of CHOP and sXBP1 (Figure 4F). These results indicate that ER stress and activation of the UPR is directly influenced by stimulation with TNFα and its activity further modulates VCAM-1 expression as a function of the relative atherogenicity of TGRL. 

### IRF-1-regulation of VCAM-1 expression correlates with TGRL-induced ER stress response

**Figure 5:**
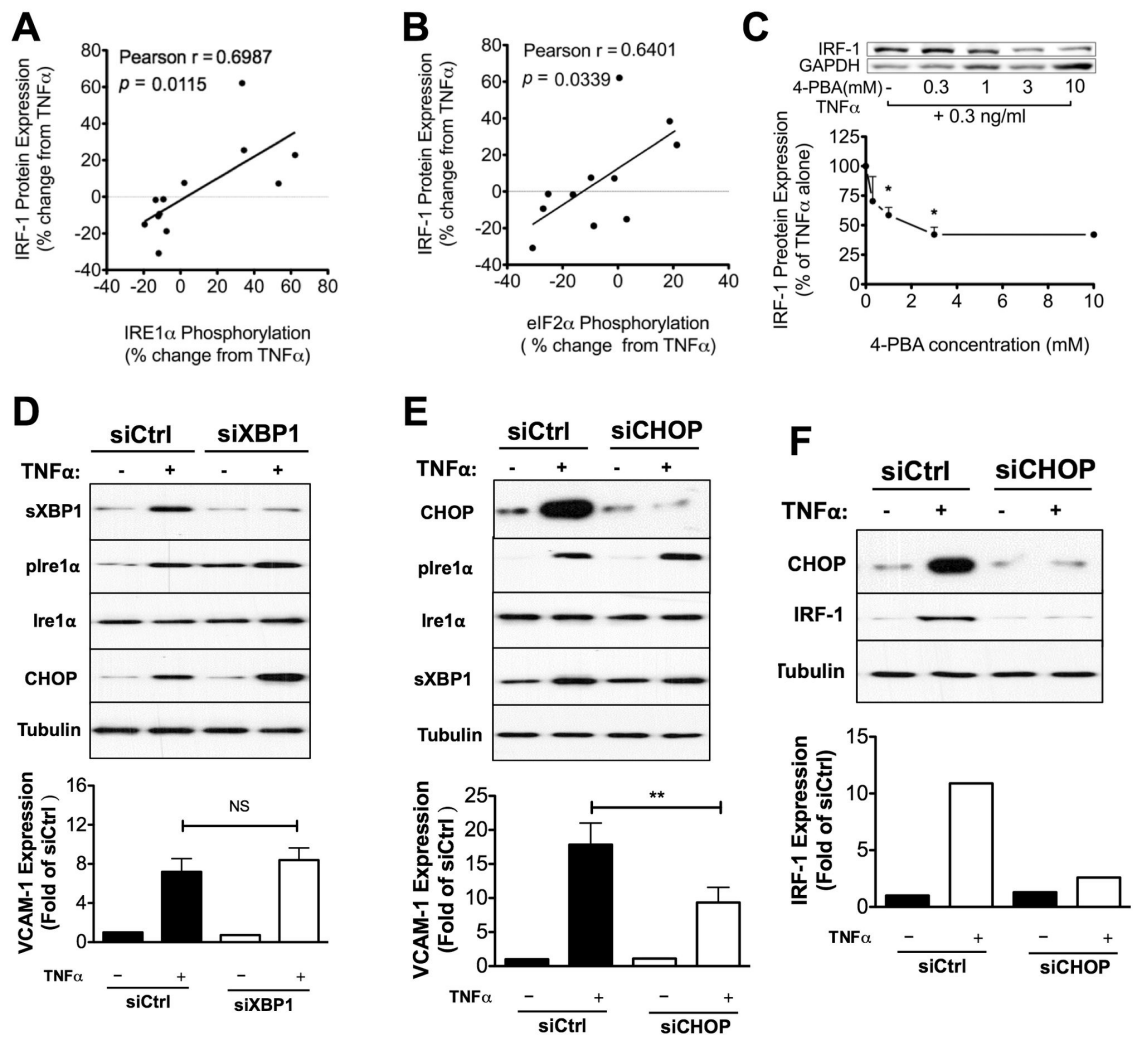
IRF-1 plays a central role in ER stress-mediated modulation of VCAM-1 expression by TGRL. A-B: TGRL modulation of TNFα-induced VCAM-1 expression is positively correlated with IRF-1 expression. TGRL modulation of TNFα-induced IRF-1 expression positively correlated with the phosphorylation levels of (A) IRE1α and (B) eIF2α. n=11-12. (C) ER stress inhibition reduced TNFα-induced IRF-1 expression in a dose-dependent manner. HAEC were conditioned for 4 hr with TNFα (0.3 ng/ml) and 4-PBA over a dose range (0-10 mM, with 1 hr pretreatment). IRF-1 protein level characterized by Western blot. Representative blots are shown. n=3-5. *P<0.05 from TNFα stimulated. D-E: CHOP knockdown (E) and not sXBP1 knockdown (D) decreased TNFα-induced VCAM-1 expression. n=4. **P<0.01 vs. siCtrl+TNFα. (F) CHOP knockdown decreases VCAM-1 expression concomitantly with an inhibition on IRF-1 expression. Shown are representative blots from 3 independent experiments with similar results.

To connect TGRL-modulated ER stress pathways to altered VCAM-1 expression, we analyzed changes in the expression of interferon regulatory factor 1 (IRF-1) with concomitant changes in ER stress markers over a range in activity from anti- to pro-atherogenic. The motivation for this set of experiments was our recent report that IRF-1 parallels inflammatory changes in VCAM-1 expression as a function of the atherogenicity of a subject’s TGRL [5]. Moreover, studies with siRNA have revealed that IRF-1 activity directly correlated with the up or down modulation of TGRL on VCAM-1 expression by regulating *Vcam-1* transcription [5]. In the current cohort, we confirmed that IRF-1 expression strongly correlated with regulation of VCAM-1 expression in response to TGRL (Figure S7 A). Like VCAM-1 expression, IRF-1 protein levels in HAEC were directly and significantly correlated with IRE1α and eIF2α phosphorylation as modulated by TGRL from our subject pool (Figure 5A-B). TGRL that promoted sXBP1 upregulation by 20% and CHOP by 24% in response to TNFα, increased IRF-1 expression on average by 23%. Conversely, TGRL that suppressed sXBP1 upregulation by 63% and CHOP by 43%, reduced IRF-1 expression by 28%. The specificity in regulation of IRF-1 was assessed by pretreatment of HAEC over a dose range of 4-PBA, which revealed a decrease in TNFα-stimulated IRF-1 protein expression stimulated with TNFα for 4 hr (Figure 5C). Similar to inhibition of VCAM-1, we detected an IC_50_ of ~1 mM and maximal suppression of IRF-1 protein production of 60% above 3mM (Figure 5C). Consistently, pretreatment of cells with 4-PBA at 1 mM significantly inhibited *Irf-1* transcription at 1 hr post stimulation with TNFα (Figure S8). Depletion of IRF-1 with siRNA decreased TNFα-induced VCAM-1 expression at both mRNA and protein levels (Figure S7 B-C). In contrast, activation of NF-κB and AP-1, two other transcription factors of *Vcam-1* that are activated by TNFα stimulation, was not affected by 4-PBA up to doses of 1 mM (Figure S9). This is consistent with our observation that ICAM-1 expression, which is primarily regulated by NF-κB and AP-1, is not significantly altered by 4-PBA treatment (Figure 3A-B). We conclude that IRF-1 activity is coupled to an ER stress response pathway that links TGRL metabolism with inflammatory regulation of VCAM-1 in HAEC. 

### CHOP is involved in the regulation of VCAM-1 expression via modulation of IRF-1

To specify the contribution of ER stress to VCAM-1 expression, the UPR downstream effectors sXBP1 and CHOP were depleted via transfection of siRNA. Western blotting confirmed successful specific knockdown of either sXBP1 or CHOP without affecting phosphorylation of IRE1α in response to TNFα stimulation (Figure 5D-E). We observed that siRNA suppression of CHOP but not sXBP1 knockdown decreased VCAM-1 expression (Figure 5D-E), concomitant with a reduction in IRF-1 expression (Figure 5F). These data are consistent with the higher propensity of TGRL to enhance CHOP expression and implicate it in activation of a pathway leading from ER stress to regulation of VCAM-1 expression. 

## Discussion

To elucidate the mechanisms that contribute to the relative atherogenicity of lipoproteins in the circulation of individuals who consume a standard high-fat meal, we exposed endothelial cells derived from human aortas to isolated postprandial TGRL. Using this model, we recently reported that TGRL from hypertriglyceridemic subjects exerted a pro-atherogenic effect on HAEC based upon its capacity to up-regulate VCAM-1 expression during inflammation [4]. In contrast, subjects with normal triglycerides produced postprandial TGRL that elicited a significant decrease in TNFα-induced VCAM-1 on HAEC [5]. Editing of VCAM-1 expression by TGRL was precisely controlled at the transcriptional level by IRF-1 and was of sufficient magnitude to significantly alter monocyte adhesion to HAEC [5]. In the current study we show that perturbation of ER function plays a central role in the modulation of VCAM-1 expression by TGRL. A direct correlation was measured between ER stress markers and the regulation of VCAM-1 in response to TGRL. Relief of ER stress with the pharmacological inhibitor 4-PBA abrogated the pro-atherogenic effect of TGRL by inhibiting IRF-1. Specifically, the anti-atherogenic effect of TGRL was recapitulated by depletion of UPR downstream effector CHOP, but not sXBP1. These data are the first to demonstrate the modulation of ER stress by native TGRL in EC and reveal that dietary lipids can differentially regulate the kinetics of TNFα-induced VCAM-1 expression at least in part through ER stress signaling and downstream pathways. 

VCAM-1 is typically expressed at undetectable levels on confluent monolayers of HAEC in tissue culture and treatment with TGRL alone does not elicit its upregulation [4]. However, the inflammatory response of HAEC to TNFα was modulated by treatment with postprandial TGRL and its atherogenic effect was recently reported to increase with particle density of cholesterol, triglycerides, ApoE and ApoCIII [5]. In the current study, we observed that TGRL uptake and the extent of LD formation was independent of TGRL atherogenicity. In light of our previous report that pro- and anti-atherogenic TGRL are composed of distinct distributions of fatty acids [5], these data suggest that the fatty acid and apolipoprotein content of TGRL and its metabolism in the ER are in part responsible for its distinct effects on inflammation. Following internalization, fatty acids and sterols from TGRL metabolism is converted to neutral lipids and stored in LD. In addition, fatty acids released from TGRL may influence ER biosynthesis of phospholipids and be incorporated into ER and LD membranes. The level of induction of ER stress appears to be related to the nature of the lipid species. Fatty acid chain length, level of unsaturation, position of double bonds, and the cis *versus* trans nature of the isomers, can all affect lipid-induced disruption of ER homeostasis and chronic ER stress [33]. For example, saturated fatty acids (SFA) such as palmitic acid can distort the composition of lipids constituting the ER membrane thereby inducing ER swelling and expansion [30,31,34,35]. In contrast, monounsaturated fatty acids (MUFA), such as oleic acid, do not induce ER stress, but are able to alleviate and reverse ER expansion and ER stress induced by SFA [30,35,36].

We observed that TNFα stimulation alone induced from ~50% to 3-fold increase in BiP expression, IRE1α and eIF2α phosphorylation, and downstream effectors, sXBP1 and CHOP. Inhibition of TNFα-induced ER stress with 4-PBA suppressed VCAM-1 expression without affecting ICAM-1 expression, suggesting a specific regulatory role of ER stress in VCAM-1 upregulation. A noteworthy observation is that treatment with pro-atherogenic TGRL alone induced ER expansion, albeit to a lesser extent than when combined with TNFα stimulation. Yet such perturbation of ER did not result in significant elevation of all ER stress markers; only CHOP expression registered a significant 1-fold increase compared to untreated control. This is consistent with CHOP being highly inducible in response to ER stress [37], and suggests that pro-atherogenic TGRL itself may prime HAEC for an ER stress susceptive state. 

UPR signaling consists of three branches that are initiated by the ER transmembrane protein sensors: IRE1α, PERK and ATF6. Activation of ER stress sensors leads to the transcription of UPR target genes through their proximal downstream effectors, such as sXBP1, the translation initiation factor eIF2α, CHOP, and p50-ATF6. Our studies reveal that IRE1α-sXBP1 and PERK-eIF2α are two UPR branches that correlated closely with TGRL’s effect on VCAM-1 upregulation. Phosphorylated IRE1α removes a 26-base intron from XBP1 mRNA, yielding the active transcription factor, sXBP1. Although we observed that the extent of IRE1α phosphorylation and sXBP1 expression correlated directly with the relative change in VCAM-1 expression for a given pro- or anti-atherogenic TGRL, we failed to establish a causal relationship between sXBP1 and VCAM-1 expression. Knockdown of sXBP1 that greatly suppressed TNFα-induced VCAM-1 in bone marrow stromal cells [38] caused no obvious reduction in TNFα-induced endothelial VCAM-1 upregulation in our study. This discrepancy may suggest specific regulation of ER stress in different cell types. Another transcription factor induced during ER stress is CHOP, which has been reported to mediate apoptosis induction under ER stress and play a role in atherogenesis [24]. It was reported that CHOP deficiency markedly suppressed expression of inflammation and adhesion related proteins including VCAM-1, IL-6, IL-1β, and MCP-1 in macrophages, EC and smooth muscle cells, and significantly reduced atherosclerotic plaque formation in high-fat diet fed ApoE^-/-^ mice [24]. In addition, deletion of CHOP in a murine model of inflammatory bowel disease significantly lowered mRNA expression of VCAM-1 and CD11b compared with wild type mice, but had no effect on expression of other adhesion molecules [39]. In line with these results, we found anti-atherogenic TGRL significantly suppressed CHOP expression induced by TNFα. Moreover, specific knockdown of CHOP mimicked the effect observed in anti-atherogenic TGRL on VCAM-1 expression.

It was previously reported that IRF-1 functioned as a specific transcriptional regulator of VCAM-1 expression in response to TGRL [5]. The current dataset confirmed that IRF-1 protein level strongly correlated with the regulation of VCAM-1 expression by TGRL. Moreover, IRF-1 expression directly correlated with TGRL-modulated eIF2α phosphorylation and was specifically regulated by the downstream effector CHOP, indicating that the PERK-eIF2α-CHOP pathway is involved in IRF-1 regulation. To analyze a link between this pathway and VCAM-1 transcription, we performed an *in silico* bioinformatics analysis of transcription factor binding sites on the promoter region of *Irf-1* using MatInspector from Genomatix. This revealed two putative CHOP binding sites. Analysis of the *Vcam-1* promoter region also revealed two putative CHOP sites. These findings suggest that CHOP may regulate VCAM-1 expression by altering IRF-1 transcription or by directly binding to the *Vcam-1* promoter region. While the results that 4-PBA decreases IRF-1 and VCAM-1 transcription support this possibility, future experimental analysis of CHOP binding to IRF-1 and VCAM-1 promoters using a CHiP assay will be necessary to elucidate the transcriptional mechanisms that function downstream of UPR pathways.

In summary, we reveal a putative mechanism by which dietary lipids can modulate inflammatory activation of VCAM-1 expression on HAEC through regulation of ER stress and UPR pathways. A systematic analysis of IRE1α, PERK/eIF2α and the proximal effector CHOP, reveal a close correlation with IRF-1 expression, which is known to regulate VCAM-1 as a function of TGRL atherogenicity. Specific knockdown with siRNA further suggest an important role of the PERK/eIF2α/CHOP pathway in IRF-1-mediated VCAM-1 expression. Inhibition of ER stress with 4-PBA greatly suppressed the increased VCAM-1 expression induced by pro-atherogenic TGRL, but only moderately lowered TGRL’s anti-atherogenic activity, suggesting that therapeutics targeting ER stress may provide efficacious treatment of atherosclerosis.

## Supporting Information

Figure S1 **TGRL atherogenicity correlates with its particle contents.** TGRL atherogenicity, defined as the ability to positively (pro-) or negatively (anti-) modulate TNFα (0.3 ng/ml)-induced VCAM-1 expression in HAEC at 4 hr, correlated with its particle contents: A) Triglyceride, B) Cholesterol, C) ApoE, and D) ApoCIII. Linear regression to data for n=13-16. Data were a representative subset of pro- and anti-atherogenic subjects, consisting of an approximately equal number of males and females.
(JPG)

Figure S2 **Cellular lipid droplet content measured by BODIPY correlates with the side scatter (SSC) signal by flow cytometry.** HAEC were treated with control media or TGRL at 10 mg ApoB/dL for periods varying from 15 min up to 8 hr, and were then washed and stained with BODIPY. The side scatter (SSC) profile and fluorescence from cells were measured by flow cytometry. Pearson correlation. MFI, median fluorescence intensity.
(JPG)

Figure S3 **Pro-atherogenic TGRL-induced ER morphological changes are exacerbated in the presence of TNFα.** HAEC grown on coverslips were treated for 4 hr with pro-atherogenic TGRL (denoted as “pro-”, 10 mg ApoB/dL) or control media in the presence or absence of TNFα at 0.3 ng/ml. Cells were then visualized by indirect immunofluorescence and confocal microscopy. Representative confocal images shown in A: red, calreticulin; blue, Hoechst nuclear stain. Bottom panels show zoomed images of calreticulin signal from the boxed 20µm x 5µm region. The extent of ER alterations was evaluated by spatial heterogeneity (standard deviation/mean of pixel fluorescence intensity within cell cytoplasm). Statistical analysis of ER expansion shown in B. n= 20-35. ***P<0.001, *P<0.05 from non-stimulated.
(JPG)

Figure S4 **Representative FACS histograms and CDF graphs of fluorescence intensity of VCAM-1 and ICAM-1 in response to TNFα and PBA.** ER stress inhibition dose-dependently reduced TNFα induced VCAM-1 expression (A-B) but did not affect ICAM-1 expression (C-D). HAEC were conditioned for 4 hr with TNFα (0.3 ng/ml) and 4-phenyl butyric acid (PBA) at different doses (0, 0.3, 1, or 3 mM, with 1 hr pretreatment).
(JPG)

Figure S5 **TNFα dose effect on ER stress response.** HAEC were treated with TNFα at 0, 0.3 or 10 ng/ml for 4 hr prior to Western Blot analysis for BiP expression (A) and IRE1α phosphorylation (B). n=3 *P<0.05; **P<0.01 vs. non-stimulated.
(JPG)

Figure S6 **TGRL modulates sXBP1 and CHOP as a function of its atherogenicity.** HAEC were treated for 4 hr with TNFα alone or simultaneously with TGRL (10 mg ApoB/dL). Pearson correlations between TGRL modulation of TNFα-induced VCAM-1 expression and (A) sXBP1 and (B) CHOP expression.
(JPG)

Figure S7 **IRF-1 mediates TGRL modulation of TNFα-induced VCAM-1 expression.** (A) TGRL modulation of TNFα-induced VCAM-1 expression correlates with IRF-1 expression. HAEC were treated for 4 hr with TNFα alone or simultaneously with TGRL (10 mg ApoB/dL). VCAM-1 membrane expression analyzed by flow cytometry and IRF-1 by Western blot. Protein expression levels presented as % change from TNFα-stimulation. Pearson correlation. (B-C) Knockdown of IRF-1 decreases VCAM-1 expression at both protein and mRNA levels. HAEC were transfected with control (siCtrl) or IRF-1 siRNA. At 48-72 hr post-transfection, cells were treated with TNFα (0.3 ng/ml). VCAM-1 expression was analyzed with quantitative RT-PCR for mRNA level (B, n=4), or flow cytometry analysis for VCAM-1 cell surface expression (C, n=4).
(JPG)

Figure S8 **Relief of ER stress with 4-PBA inhibits IRF-1 transcription.** HAEC were pretreated with 1mM 4-phenyl butyric acid (4-PBA) for 1 hr followed by stimulation with 0.3 ng/mL TNFα for 30 min or 1 hr. IRF-1 mRNA was quantified with quantitative RT-PCR. Paired Student’s *t* test. *P<0.05.
(JPG)

Figure S9 **4-PBA does not affect TNFα-induced NF-κB and AP-1 activity.** Activation of (A) NF-κB (phospho-p65) and (B) AP-1 (phosphorylated c-Jun) was measured by ELISA in nuclear extract of HAEC treated for 30 min with 4-PBA over a dose range of 0-10 mM simultaneously with TNFα (0.3 ng/ml). (A) n=3-6. ns, not significant. RLU, relative light unit. (B) n=4-6. *P<0.05; **P<0.01 vs. TNFα stimulated.
(JPG)

Table S1 **Anthropometric characteristics of study participants.**
(DOCX)

Table S2 **Fasting and postprandial glucose and cholesterol levels of subjects.**
(DOCX)
