# Expression of Concern: Triglyceride-Rich Lipoprotein Modulates Endothelial Vascular Cell Adhesion Molecule (VCAM)-1 Expression via Differential Regulation of Endoplasmic Reticulum Stress

**DOI:** 10.1371/journal.pone.0300609

**Published:** 2024-03-13

**Authors:** The PLOS ONE Editors

Following the publication of this article [1], concerns were raised regarding results presented in Figs 4 and 5. Specifically,

- In the Fig 4B p-Ire1α panel, there appears to be a vertical discontinuity suggestive of a splice line between lanes 5 and 6.
- The following panels appear similar, despite being used to represent different experimental conditions:
  ○ The Fig 4F pIre1α panel and the Fig 4F CHOP panel with aspect ratio altered.
  ○ Lanes 1–3 of the Fig 5D sXBP1 panel and lanes 1–3 of the Fig 5D CHOP panel, when one of the panels is rotated 180°.
  ○ The Fig 4F Tubulin panel and the Fig 5D Tubulin panel with aspect ratio altered.

The corresponding author stated that the western blot for Fig 4B p-Ire1α was in no way altered or processed differently from the other blots presented in the article. Furthermore, the corresponding author disagreed with the concerns regarding similarity between panels presented in Figs 4F and 5D, and stated that the original data underlying these results are no longer available due to the time elapsed since the submission of the article. Although the original images underlying the published panels are no longer available, the corresponding author provided the individual level data underlying the graphs presented in Fig 5D–5F. These individual level underlying data have been made available in the S1 File with this notice. In the absence of the original image data underlying the published figures, the concerns with Figs 4B, 4F and 5D cannot be resolved.

In light of the unresolved image concerns, the following findings reported in this study are not sufficiently supported:

- Pro-atherogenic TGRL increased the phosphorylation of IRE1α (35%) from the modest effect of TNFα alone, whereas anti-atherogenic TGRL had comparatively less of an effect on IRE1α.
- Inhibition with 4-PBA down regulated cytokine activation of ER stress as indicated by an attenuated phosphorylation of IRE1α and reduced expression of CHOP.
- Western blotting confirmed successful specific knockdown of sXBP1 without affecting phosphorylation of IRE1α in response to TNFα stimulation.
- siRNA suppression of sXBP1 knockdown did not decrease VCAM-1 expression.

The *PLOS ONE* Editors issue this Expression of Concern to notify readers of the above concerns. In light of these concerns, the results presented in Figs 4B, 4F and 5D, and conclusions respective to these results should be interpreted with caution.

## Supporting information

S1 File Individual level data underlying graphs presented in Fig 5D–5F. (DOCX)
