# Supplementary figures and images for: Triglyceride-Rich Lipoprotein Modulates Endothelial Vascular Cell Adhesion Molecule (VCAM)-1 Expression via Differential Regulation of Endoplasmic Reticulum Stress
Source: PLoS One. 2013 Oct 21;8(10):e78322. doi: 10.1371/journal.pone.0078322 (PMC3804477; doi:10.1371/journal.pone.0078322)

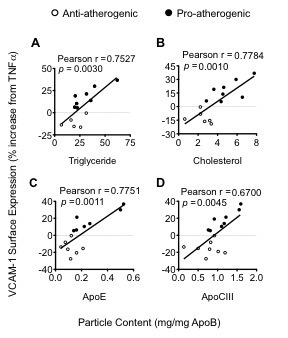

Supplement: Figure S1 — TGRL atherogenicity correlates with its particle contents. TGRL atherogenicity, defined as the ability to positively (pro-) or negatively (anti-) modulate TNFα (0.3 ng/ml)-induced VCAM-1 expression in HAEC at 4 hr, correlated with its particle contents: A) Triglyceride, B) Cholesterol, C) ApoE, and D) ApoCIII. Linear regression to data for n=13-16. Data were a representative subset of pro- and anti-atherogenic subjects, consisting of an approximately equal number of males and females. (JPG) [file pone.0078322.s001.jpg]

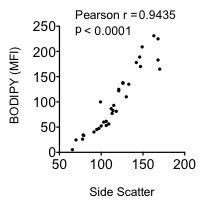

Supplement: Figure S2 — Cellular lipid droplet content measured by BODIPY correlates with the side scatter (SSC) signal by flow cytometry. HAEC were treated with control media or TGRL at 10 mg ApoB/dL for periods varying from 15 min up to 8 hr, and were then washed and stained with BODIPY. The side scatter (SSC) profile and fluorescence from cells were measured by flow cytometry. Pearson correlation. MFI, median fluorescence intensity. (JPG) [file pone.0078322.s002.jpg]

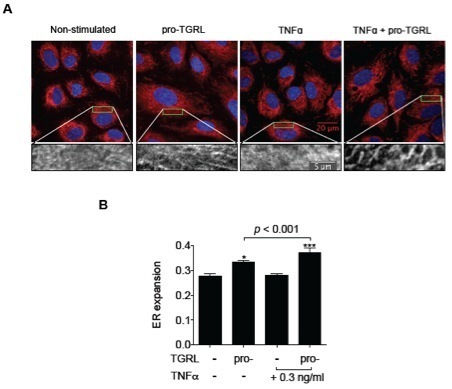

Supplement: Figure S3 — Pro-atherogenic TGRL-induced ER morphological changes are exacerbated in the presence of TNFα. HAEC grown on coverslips were treated for 4 hr with pro-atherogenic TGRL (denoted as “pro-”, 10 mg ApoB/dL) or control media in the presence or absence of TNFα at 0.3 ng/ml. Cells were then visualized by indirect immunofluorescence and confocal microscopy. Representative confocal images shown in A: red, calreticulin; blue, Hoechst nuclear stain. Bottom panels show zoomed images of calreticulin signal from the boxed 20µm x 5µm region. The extent of ER alterations was evaluated by spatial heterogeneity (standard deviation/mean of pixel fluorescence intensity within cell cytoplasm). Statistical analysis of ER expansion shown in B. n= 20-35. ***P<0.001, *P<0.05 from non-stimulated. (JPG) [file pone.0078322.s003.jpg]

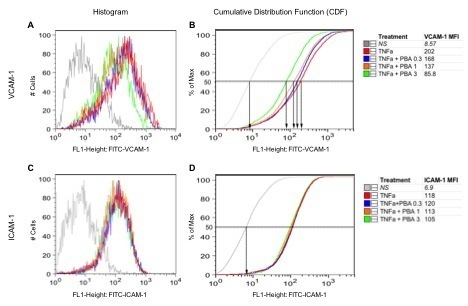

Supplement: Figure S4 — Representative FACS histograms and CDF graphs of fluorescence intensity of VCAM-1 and ICAM-1 in response to TNFα and PBA. ER stress inhibition dose-dependently reduced TNFα induced VCAM-1 expression (A-B) but did not affect ICAM-1 expression (C-D). HAEC were conditioned for 4 hr with TNFα (0.3 ng/ml) and 4-phenyl butyric acid (PBA) at different doses (0, 0.3, 1, or 3 mM, with 1 hr pretreatment). (JPG) [file pone.0078322.s004.jpg]

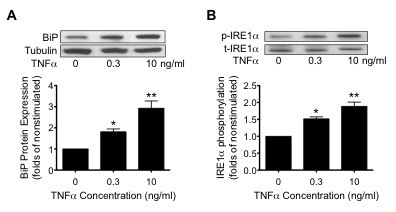

Supplement: Figure S5 — TNFα dose effect on ER stress response. HAEC were treated with TNFα at 0, 0.3 or 10 ng/ml for 4 hr prior to Western Blot analysis for BiP expression (A) and IRE1α phosphorylation (B). n=3 *P<0.05; **P<0.01 vs. non-stimulated. (JPG) [file pone.0078322.s005.jpg]

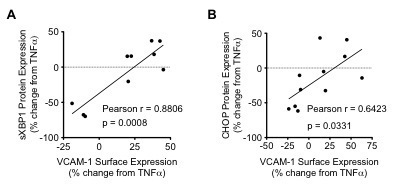

Supplement: Figure S6 — TGRL modulates sXBP1 and CHOP as a function of its atherogenicity. HAEC were treated for 4 hr with TNFα alone or simultaneously with TGRL (10 mg ApoB/dL). Pearson correlations between TGRL modulation of TNFα-induced VCAM-1 expression and (A) sXBP1 and (B) CHOP expression. (JPG) [file pone.0078322.s006.jpg]

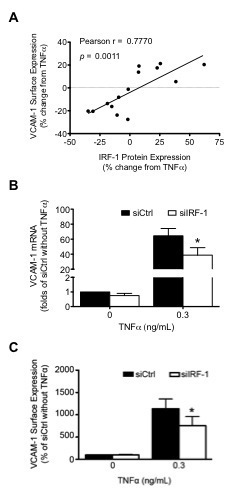

Supplement: Figure S7 — IRF-1 mediates TGRL modulation of TNFα-induced VCAM-1 expression. (A) TGRL modulation of TNFα-induced VCAM-1 expression correlates with IRF-1 expression. HAEC were treated for 4 hr with TNFα alone or simultaneously with TGRL (10 mg ApoB/dL). VCAM-1 membrane expression analyzed by flow cytometry and IRF-1 by Western blot. Protein expression levels presented as % change from TNFα-stimulation. Pearson correlation. (B-C) Knockdown of IRF-1 decreases VCAM-1 expression at both protein and mRNA levels. HAEC were transfected with control (siCtrl) or IRF-1 siRNA. At 48-72 hr post-transfection, cells were treated with TNFα (0.3 ng/ml). VCAM-1 expression was analyzed with quantitative RT-PCR for mRNA level (B, n=4), or flow cytometry analysis for VCAM-1 cell surface expression (C, n=4). (JPG) [file pone.0078322.s007.jpg]

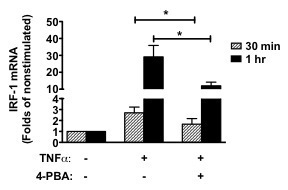

Supplement: Figure S8 — Relief of ER stress with 4-PBA inhibits IRF-1 transcription. HAEC were pretreated with 1mM 4-phenyl butyric acid (4-PBA) for 1 hr followed by stimulation with 0.3 ng/mL TNFα for 30 min or 1 hr. IRF-1 mRNA was quantified with quantitative RT-PCR. Paired Student’s t test. *P<0.05. (JPG) [file pone.0078322.s008.jpg]

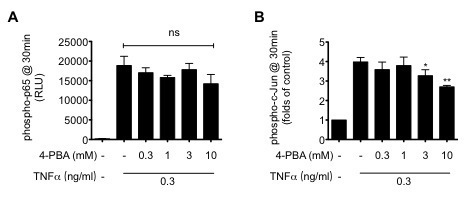

Supplement: Figure S9 — 4-PBA does not affect TNFα-induced NF-κB and AP-1 activity. Activation of (A) NF-κB (phospho-p65) and (B) AP-1 (phosphorylated c-Jun) was measured by ELISA in nuclear extract of HAEC treated for 30 min with 4-PBA over a dose range of 0-10 mM simultaneously with TNFα (0.3 ng/ml). (A) n=3-6. ns, not significant. RLU, relative light unit. (B) n=4-6. *P<0.05; **P<0.01 vs. TNFα stimulated. (JPG) [file pone.0078322.s009.jpg]
