# Supplementary material for: Triglyceride-Rich Lipoprotein Modulates Endothelial Vascular Cell Adhesion Molecule (VCAM)-1 Expression via Differential Regulation of Endoplasmic Reticulum Stress
Source: PLoS One. 2013 Oct 21;8(10):e78322. doi: 10.1371/journal.pone.0078322 (PMC3804477; doi:10.1371/journal.pone.0078322)
Supplement: Table S1 — Anthropometric characteristics of study participants. (DOCX) [file pone.0078322.s010.docx]

**Table S1: Anthropometric characteristics of study participants**

|  | N =34* |
| --- | --- |
| Age, years | 37.3 ± 14.3 |
| BMI, kg/m^2^ | 28.3 ± 6.7 |
| WHR | 0.91 ± 0.07 |
| Waist circumference, m | 0.86 ± 0.14 |

*18 males and 16 females. BMI, body mass index; WHR, waist to hip ratio; Mean ± S.D.
