# Supplementary material for: Triglyceride-Rich Lipoprotein Modulates Endothelial Vascular Cell Adhesion Molecule (VCAM)-1 Expression via Differential Regulation of Endoplasmic Reticulum Stress
Source: PLoS One. 2013 Oct 21;8(10):e78322. doi: 10.1371/journal.pone.0078322 (PMC3804477; doi:10.1371/journal.pone.0078322)
Supplement: Table S2 — Fasting and postprandial glucose and cholesterol levels of subjects. (DOCX) [file pone.0078322.s011.docx]

**Table S2: Fasting and postprandial glucose and cholesterol levels of subjects**

|  | **Fasting, mg/dL** | **Postprandial, mg/dL** |
| --- | --- | --- |
| Triglycerides | 125.3 ± 76.5 | 259.4 ± 173.6 *** |
| Glucose | 90.4 ± 6.4 | 84.9 ± 10.0 * |
| Total cholesterol | 192.9 ± 48.0 | 191.6 ± 44.2 |
| HDL cholesterol | 51.9 ± 17.0 | 49.8 ± 15.0 * |
| LDL cholesterol | 114.7 ± 43.8 | 94.3 ± 39.5 *** |
| Apolipoprotein B100 | 89.3 ± 31.8 | 89.7 ± 32.6 |

Mean ± S.D. * p<0.05, *** p<0.001 from fasting by paired Student’s *t* test.
