# Supplementary material for: Expression of Concern: Triglyceride-Rich Lipoprotein Modulates Endothelial Vascular Cell Adhesion Molecule (VCAM)-1 Expression via Differential Regulation of Endoplasmic Reticulum Stress
Source: PLoS One. 2024 Mar 13;19(3):e0300609. doi: 10.1371/journal.pone.0300609 (PMC10936785; doi:10.1371/journal.pone.0300609)
Supplement: S1 File — (DOCX) [file pone.0300609.s001.docx]

**Triglyceride-Rich Lipoprotein Modulates Endothelial Vascular Cell Adhesion Molecule (VCAM)-1 Expression via Differential Regulation of Endoplasmic Reticulum Stress, Wang et al, 2013.**

**Data corresponding to Figures 5 D-F**


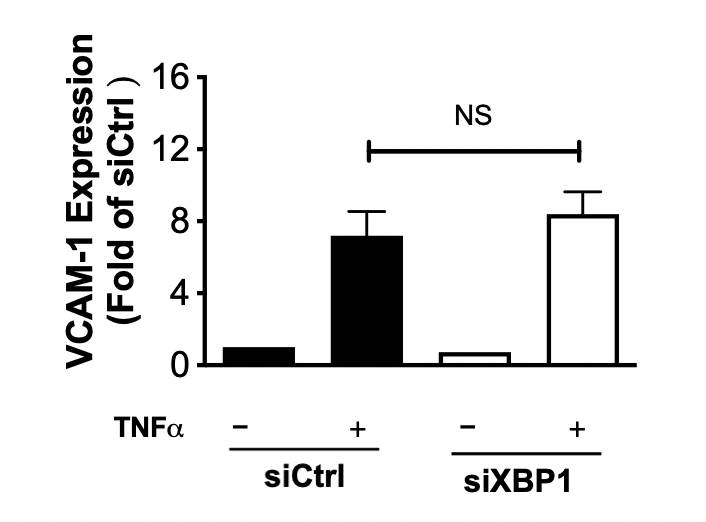
Fig 5D

| siCtrl | siCtrl+TNFa | siXBP1 | siXBP1+TNFa |
| --- | --- | --- | --- |
| 1 | 9.89 | 0.73 | 10.19 |
|  | 5.85 |  | 8.99 |
|  | 5.85 |  | 6.00 |

Fig 5E


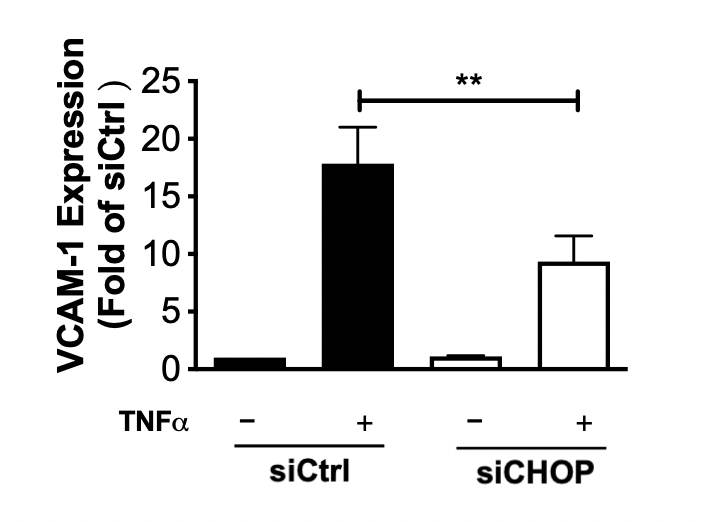


| siCtrl | siCHOP | siCtrl+TNFa | siCHOP+TNFa |
| --- | --- | --- | --- |
| 1 | 1.09 | 10.89 | 7.16 |
| 1 | 1.16 | 19.36 | 9.95 |
| 1 | 1.00 | 25.79 | 15.31 |
| 1 | 1.23 | 15.33 | 4.97 |


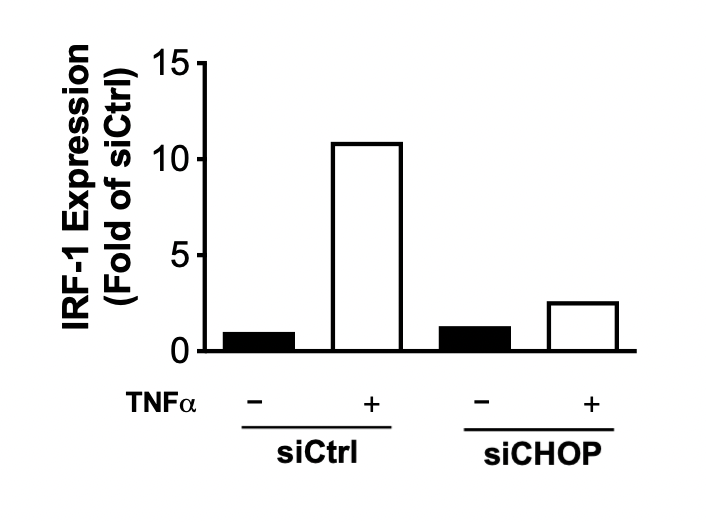
Fig 5F

| siCtrl | siCtrl+TNFa | siCHOP | siCHOP+TNF |
| --- | --- | --- | --- |
| 1 | 10.9 | 1.3 | 2.6 |
